# High Temperature AlGaN/GaN Membrane Based Pressure Sensors

**DOI:** 10.3390/mi9050207

**Published:** 2018-04-28

**Authors:** Durga Gajula, Ifat Jahangir, Goutam Koley

**Affiliations:** 1 Holcombe Department of Electrical and Computer Engineering, Clemson University, Anderson, SC 29625, USA; gkoley@clemson.edu; 2 Department of Electrical Engineering, University of South Carolina, Columbia, SC 29208, USA; ifat00@gmail.com

**Keywords:** MEMS, high temperature pressure sensors, AlGaN/GaN circular HFETs, GaN diaphragm

## Abstract

A highly sensitive Gallium Nitride (GaN) diaphragm based micro-scale pressure sensor with an AlGaN/GaN heterostructure field effect transistor (HFET) deflection transducer has been designed and fabricated for high temperature applications. The performance of the pressure sensor was studied over a pressure range of 20 kPa, which resulted in an ultra-high sensitivity of ~0.76%/kPa, with a signal-to-noise ratio as high as 16 dB, when biased optimally in the subthreshold region. A high gauge factor of 260 was determined from strain distribution in the sensor membrane obtained from finite element simulations. A repeatable sensor performance was observed over multiple pressure cycles up to a temperature of 200 °C.

## 1. Introduction

In harsh environments, such as in the aerospace, automotive, nuclear power and petroleum industries, there is a great need for high temperature pressure sensors [1,2,3]. Silicon electrical properties degrade with the temperatures above 150 °C, due to the generation of thermal carriers and high leakage currents, which makes it less suitable for harsh environments. The corrosion resistance of silicon is also limited at high temperatures. This moved the researcher’s interest to higher bang-gap materials like SiC, AlN, Gallium Nitride (GaN) and so forth [4,5,6,7,8,9,10,11]. Due to their higher band gap, these materials have excellent thermal stability at higher temperatures. Among these, AlGaN got a special interest due to its excellent piezo-electric properties [12]. It is also chemically inert, mechanically stable and radiation hardened, which makes it a promising device material for hostile environments. GaN layer is highly piezo electric and AlGaN/GaN heterostructure have a spontaneous polarization at the interface, which creates a 2DEG (two-dimensional electron gas) at the interface [13]. This 2DEG offers a great opportunity for using AlGaN/GaN in piezo-resistive and piezo-electric transducers, since both the 2DEG density and the mobility of the 2DEG can be modulated with the strain. The sensitivity for the applied strain for AlGaN/GaN transducers will have higher values than those of silicon, where only the carrier mobility gets modulated with the applied strain. These characteristics of AlGaN/GaN interface make them better suitable material for the sensing applications over silicon [7,8,9,10,11,12,13,14,15]. Silicon carbide based piezo resistive pressure sensors at temperatures up to 600 °C have been studied, however these devices have low output signals and low pressure sensitivity values [16,17,18]. Capacitive sensing is a dominant technique in pressure sensing, however the motion of the sensor is constrained to a small vertical and horizontal movements [19]. If the vertical displacement is large, the capacitance are not suitable for pressure sensing. So, the interest has been shifted to diaphragm based pressure sensors, where the sensors motion is dependent on the yield strength of the material than on the design constraints. The theoretical temperature limit of Gallium Nitride (GaN) can be as high as 600 °C and GaN heterostructure also shown higher mobility values and high critical breakdowns [20]. The III-V nitrides is also having a high potential for monolithic integration [21]. There have been various studies on the AlGaN/GaN based pressure sensors for room temperature sensing applications [22,23]. In this article, we have investigated high temperature pressure sensing behavior of AlGaN/GaN based devices and its electrical properties with the applied pressure have been studied with the temperature. The mechanical stress distribution across the circumference of the diaphragm with pressure will provide the change in piezoelectric charge in GaN HFET (hetero-structure field-effect transistor) and so thus the change in source-drain resistance. The pressure sensitivity values are significantly better than existing technologies, which underscores the prospect of these devices for high temperature pressure sensing applications.

## 2. Materials and Methods

**Figure 1:**
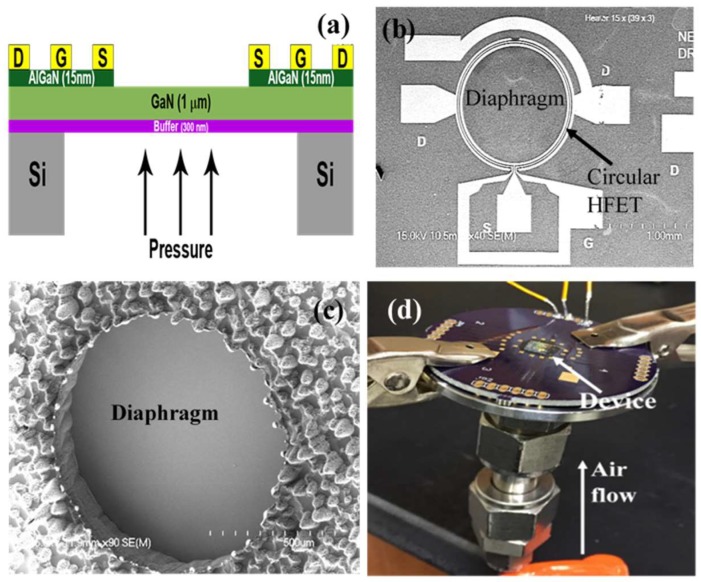
(**a**) Cross-sectional structure of AlGaN/GaN heterostructure field effect transistor (HFET) pressure sensor; (**b**) Diaphragm based AlGaN/GaN HFET pressure sensor, with a radius of 1000 µm diaphragm; (**c**) The back side of the diaphragm, where the pressure was applied; (**d**) the experimental set-up for pressure sensing.

**Figure A1:**
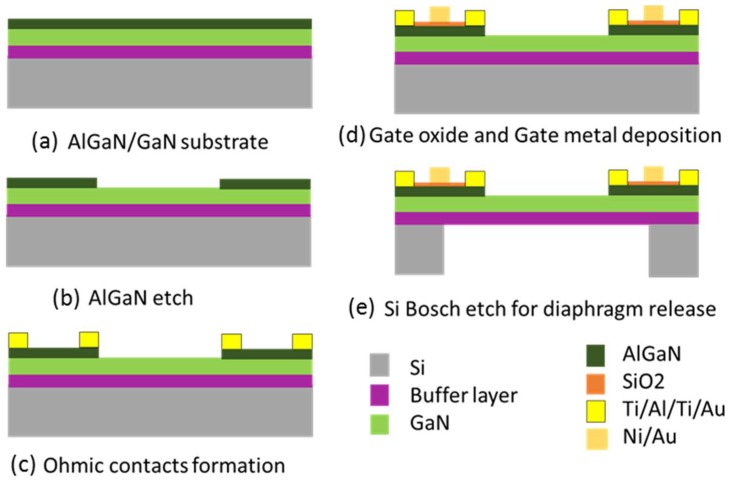
Diagrammatic representation of process flow for the fabrication of pressure sensors. (**a**)–(**e**) Different steps of the process flow shown in order, as discussed in the text.

The pressure transducers used in this study were fabricated on AlGaN/GaN epitaxial layers on (111) silicon wafer, purchased from NTT Advanced Technology Corporation, Japan. The wafer had a 2 nm i-GaN cap layer and 15 nm Al_0.25_Ga_0.75_N on top of 1 µm i-GaN, with a 300 nm buffer layer separating the GaN layer from the 675–750 µm thick Si substrate. At the beginning of the fabrication process, the top 100 nm of AlGaN/GaN layer was etched using BCl_3_/Cl_2_ plasma chemistry to define the mesa region at the periphery of the diaphragm, followed by deposition of a Ti (20 nm)/Al (100 nm)/Ti (45 nm)/Au (55 nm) metal stack. A rapid thermal annealing process was performed at 825 °C for a minute, to form ohmic contacts for the source and drain regions for the HFET. After that, plasma enhanced chemical vapor deposition (PECVD) technique was used to deposit 100 nm thick SiO_2_ to cover the open regions of the mesa, which served as the gate dielectric. This was followed by two consecutive stages of metallization, the first one had Ni (25 nm)/Au (200 nm) stack as the gate metal contacts and the second one had Ti (20)/Au (225 nm) stack to from the probe contacts. Finally Bosch process was used from the bottom face of the sample to perform through wafer etching of silicon to release the diaphragm. Figure 1a shows the schematic diagram of the pressures sensor (Appendix A: Figure A1 represents the diagrammatic representation of the process flow the fabrication of these pressure sensors). Figure 1b,c shows the scanning electron microscopic (SEM) images of a diaphragm from the topside and the backside of the sample.

## 3. Results and Discussion

The sheet resistance of the AlGaN layer and the contact resistance of the ohmic metal pads were found out to be 316 Ω/cm^2^ and 19 Ω/cm^2^ respectively, measured using transmission line measurement (TLM) technique. The transfer length, *L_T_*, was found to be 18 µm and contact resistivity was calculated as *ρ_c_* = (*L_T_*)^2^ × *R_sheet_* = 0.57 Ω·cm^2^. However, due to the device contacts being of shorter length (10 μm) than the *L_T_*, the estimated total contact resistance for both contacts is about 8.6 Ω, while the overall channel resistance is about 2.6 Ω, which means a significant amount of voltage (~77%) is dropped at the contacts, which needs to be accounted for in the calculations that follow.

For electrical and pressure transduction measurements, the HFET embedded diaphragms were glued to a printed circuit board (PCB) with a small pinhole in such a way that the pinhole is aligned with the diaphragm and the glue forms a vacuum seal. The contact pads were wire bonded and the PCB was mounted on a high pressure fixture shown in Figure 1d, where a gas line was installed beneath the diaphragm with high temperature O-rings for good vacuum sealing. A heater and a thermo-couple were also placed on top of the PCB, near the transducer chip, to carry out the experiments at higher temperatures. At first, we performed transistor measurements to calculate field effect mobility, given by

(1) $$\mu_{FET}=\frac{g_{m}\times l_{g}}{q\times V_{ds,e}\times C_{g}}$$

where *g_m_* is the drain-source transconductance, *q* is the electron charge, *l_g_* is the gate length of the channel, *C_g_* is the gate capacitance, *V_ds_*_,*e*_ is the effective voltage drop at the channel (across the intrinsic transistor), estimated as a fraction of applied *V_ds_* from the TLM measurements. Since the series combination of the oxide capacitance and the capacitance of the top AlGaN layers dominate the overall capacitance, we consider this constant capacitance as *C_g_*, without performing a full gate capacitance-voltage (C-V) measurement.

**Figure 2:**
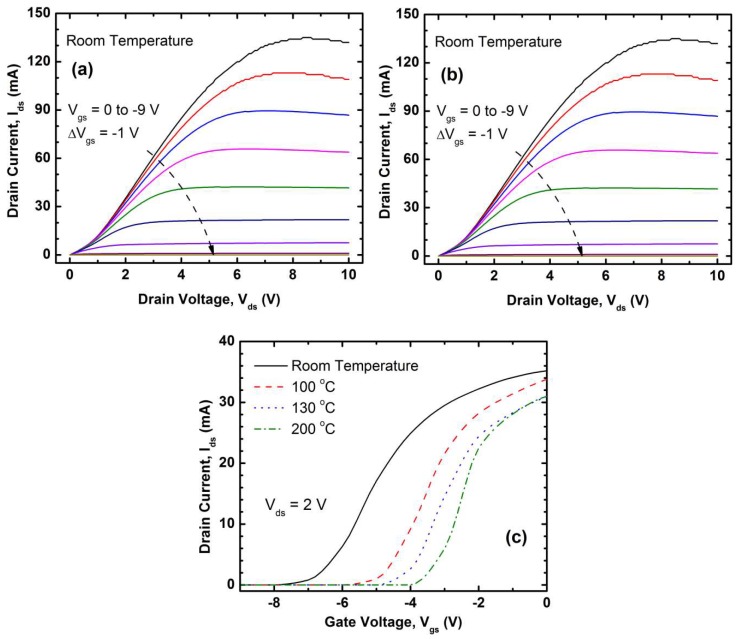
(**a**) *I_ds_*-*V_ds_* characteristics of the circular AlGaN/GaN HFET at room temperature; (**b**) variation of *I_ds_* at *V_gs_* = 0 V as a function of temperature and (**c**) *I_ds_-V_gs_* characteristics of the HFET measured at different temperatures and at *V_ds_* = 1 V.

Figure 2a shows the *I_ds_*-*V_ds_* characteristics the HFET at room temperature (RT). Here the non-linearity observed at the low bias range can be attributed to the non-ideality of the contacts that was evident from the relatively higher contact resistivity. The current eventually saturates or reduces at different *V_ds_* depending on the *V_gs_*, which is expected from a well-behaved HFET with high current driving capacity. Figure 2b shows the variation of *I_ds_* at *V_gs_* = 0 V as a function of temperature. As the temperature goes up, the peak Ids goes down and so does the corresponding *V_ds_*, as the channel resistance increases as a result of increased scattering. However, at low *V_ds_* (<1 V), the *I_ds_*-*V_ds_* curves become more linear at higher temperatures, which can be attributed to the improved thermionic emission at the non-ideal contacts. That is why we calculate the field effect mobility at a higher *V_ds_* (~V), as it is less sensitive to the Schottky-like behavior at the lower *V_ds_*. Figure 2c shows the *I_ds_-V_gs_* characteristics of the HFET measured at different temperatures and at *V_ds_* = 1 V. The peak transconductance at room temperature was ~15 mA/V, which resulted in a raw value of *µ_FET_* = ~300 cm^2^/(V·s) and sheet carrier concentration *n_s_* = 7.19 × 10^11^ cm^−2^ at *V_gs_* = −2 V and 2.35 × 10^11^ cm^−2^ at *V_gs_* = −7 V. This low mobility is likely attributable to significant voltage drop across the drain and source contact resistances due to their sub-optimal width (< transfer length) caused by spatial limitation. The true value of the mobility lies between these two extreme cases. The reduction of mobility at RT is generally associated with the enhanced defect scattering in HFET and significant carrier trapping at the 2DEG surface [24].

From Figure 2c, we observe that the threshold and turn off voltages of the HFET were reduced with the increase of temperature. The threshold voltages of HFET at RT, 100 °C, 130 °C and 200 °C are approximately −7.5 V, −5.7 V, −4.9 V and −4 V respectively. This is in good agreement with the study by Alim et al. [25] on the variation of the threshold with the temperature, where they also noticed that the positive shift in the Schottky barrier height along with trap-assisted phenomena shifted the threshold voltage towards positive values as the temperature was increased. At higher temperatures, the phonon scattering also plays a dominant role leading to the reduction in the mobility values.

**Figure 3:**
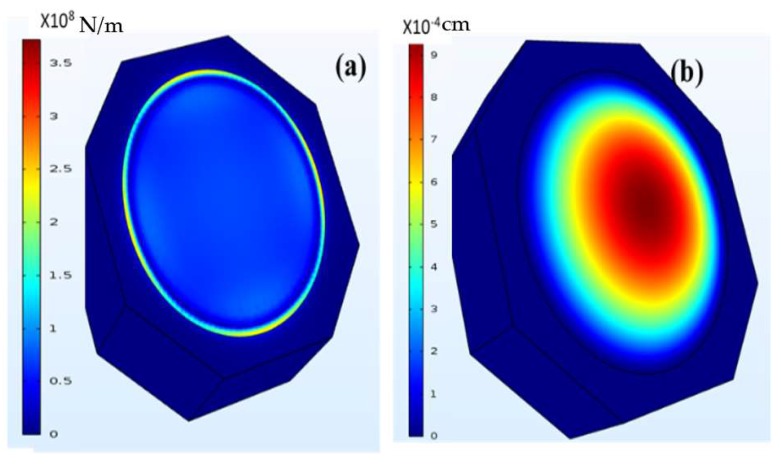
(**a**) Stress (N/m^2^) simulations results on AlGaN/GaN diaphragm using finite element method using COMSOL (**b**) the displacement (cm) of the diaphragm with applied pressure (20 kPa).

Since AlGaN/GaN heterojunction has a spontaneous piezoelectric polarization at the interface, any external strain changes the density of the mobile carriers (2DEG) at the interface. The associated change in resistance with strain can be used as a direct measure of the strain that is being applied on the system [26,27]. Figure 3 shows the finite element (FE) simulations using COMSOL Multiphysics (version 4.3, COMSOL Inc., Stockholm, Sweden), which shows the (a) stress values across the diaphragm and the (b) displacement of the diaphragm at an applied pressure of 20 kPa above atmospheric pressure. From this computation, the maximum displacement of the diaphragm was estimated to be ~10 µm. The maximum stress in the diaphragm was at the circumference and because of we designed the HFETs to be at the periphery of the diaphragm to maximize the polarization-induced change in conductivity and hence the maximum sensitivity.

**Figure 4:**
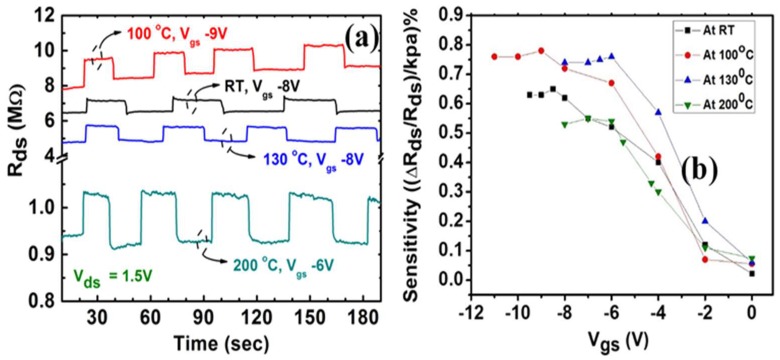
(**a**) The change in drain-source resistance, with applied pressure in regular intervals, indicated by the increased *R_ds_*. The figure shows the measurement at different temperatures, *V_ds_* is 1.5 V and pressure difference is 20 kPa; (**b**) The variation of pressure sensor sensitivity with gate voltage of HFET, at different temperature. *V_ds_* is 1.5 V and the applied differential pressure is 20 kPa.

Figure 4a shows the variation of source drain resistance (*R_ds_*) with 20 kPa of pressure difference being applied to the diaphragm in regular intervals, which resulted in the *R_ds_* increasing. The pressure was applied and released quickly using a valve to reduce mechanical transients in the measurements. At each measurement point, the differential pressure was kept at 20 kPa for few seconds and then was reduced back to zero (atmospheric pressure) and repeated the experiments for a number of cycles. This was repeated for various temperatures and the results are compared in Figure 4a, where we kept the drain source voltage at 1.5 V but varied the gate voltage to achieve the highest sensitivity for each temperature. The signal to noise ratio is calculated for each dataset using the expression, $SNR_{dB}=10\log_{10}\left(\frac{R_{signal}}{R_{noise}}\right)$, where *R_signal_* is the average change in resistance when the strain is applied and *R_noise_* is the average variation in resistance when there is no strain. The calculated signal-to-noise ratio (SNR) is15–16 dB for these pressure sensors, for all the measured temperatures. The rise and fall times of the response are ~200 ms and ~600 ms respectively, which includes the mechanical transient arising from the time required for the pressure to reach the steady-state. Therefore, actual electrical transient is negligible, which is quite extraordinary for an AlGaN/GaN HFET without any surface passivation.

The sensitivity of the device is calculated from the equation, $S=\frac{\Delta R}{R_{o}}\frac{1}{P}\%$. Here *S* is defined as the percentage change in the resistance, with respect to the pressure difference (*P*). Gauge factor, *GF* of the device can be derived as

(2) GF=ΔRR01∈=1∈(Δμnμn+Δnsns)

where *R*_0_ is the initial resistance and ∆*R* is the change in resistance with applied strain, ∈. *µ_n_* and *n_s_* are the mobility and carrier concentrations respectively. From Equation (1), we see that the gauge factor depends on the changes in mobility and carrier concertation. In an HFET device, the gate voltage can be tuned deplete the 2DEG to bring *n_s_* to a low level, also known as the subthreshold region, where a small change in *n_s_* caused by the strain can significantly affect the Δnsns ratio and increase *GF* [28]. As the temperature goes up, mobility decreases in general; but due to the imperfect contacts, the change in conductivity as a function of temperature does not follow the same trend as mobility in low *V_ds_*, which causes a non-monotonous change in device response measured at *V_ds_* = 1.5 V. In Figure 4a, we see that the response magnitude and sensitivity increase from RT to 100 °C and then gradually decrease through 200 °C. At higher temperature, *µ_n_* decreases [29], as a result, overall sensitivity is expected to decrease. However, because of our contacts being shorter than the transfer length, we have variable contact resistance which improves with slightly higher temperature (Figure 2b) due to the increased efficiency in thermionic emission process. This increases the injection efficiency from the contacts into the channel, which allows the changes in Δ*n_s_* and Δ*R* to appear larger under applied pressure as well, due to the non-linearity in the *I_ds_*-*V_ds_* curves at the low field. However, as temperature keeps on increasing, the contact resistance is expected to reach an equilibrium at one point (*L_T_* coming closer to the contact length) and the high temperature causes the sensitivity to drop.

Figure 4b, which shows the variation of the pressure sensitivity as a function of *V_gs_* of the HFET at different temperatures, is in good agreement with the aforementioned explanation. For all four temperatures shown here, the sensitivity becomes nearly constant for *V_gs_* < −6 V, which indicates that a *V_gs_* just below −6 V is the optimal gate bias. The change in *n_s_* is maximum when *V_gs_* is between 0 V and about −6 V, which is why the large changes in sensitivity is only observed in this region. At zero gate bias, very low sensitivity is observed since the baseline carrier concentration *n_s_* is very high (order of high 10^12^ cm^−2^), while the change in carrier concentration Δ*n_s_* due to deflection related strain is very low. Due to this a gate control is required to reduce the 2DEG density which will automatically increase the sensitivity (proportional to Δ*n_s_*/*n_s_*) [30]. This is in agreement with an earlier study by Zimmermann et al. which also showed that the pressure response increases at higher gate bias [31]. The sensitivity of our pressure sensors varied from 0.022%/kPa, at zero gate voltage, to 0.5–0.76%/kPa in the subthreshold region (*V_g_* ≈ −6 V, see discussion above) for different temperatures, with the maximum gauge factor (GF) being ~260. The corresponding sensitivity in terms of change in the drain voltage (assuming a constant current of ≈1 × 10^−7^ amp) is ~7–18 mV/kPa, which is slightly higher than the value of 7.25–14.5 mV/kPa reported for commercial high sensitivity pressure sensors (IMI sensors) [32]. It is important to note that the sensitivity values obtained from our AlGaN/GaN pressure sensor are orders of magnitude higher than the sensitivity value of 0.02% change for 50 bar reported by Boulbar et al. on AlGaN/GaN heterojunction based pressure sensors fabricated on sapphire substrate [19]. Our results are also close to an order of magnitude better than the recently reported sensitivity value of 0.64%/psig (= 0.09%/kPa) measured on InAlN/GaN heterostructure based micro-pressure sensors [33].

## 4. Conclusions

In summary, we have demonstrated for the first time a diaphragm based AlGaN/GaN HFET embedded circular membrane pressure sensor for high temperature pressure sensing, with ultra-high sensitivity. Finite element simulation was utilized to determine the strain across the diaphragm and determine the gauge factor, which was found out to be ~260 in the sub-threshold region. A very high sensitivity of 0.76%/kPa was also measured, which is the highest reported so far for III-Nitride based pressure sensors. The pressure sensor performance was found to be quite repeatable and was maintained up to a temperature of 200 °C.
